# On the optimization of bone SPECT/CT in terms of image quality and radiation dose

**DOI:** 10.1002/acm2.13069

**Published:** 2020-10-27

**Authors:** Monika Tulik, Piotr Tulik, Teresa Kowalska

**Affiliations:** ^1^ Maria Sklodowska‐Curie National Research Institute of Oncology Krakow Branch Krakow Poland; ^2^ Warsaw University of Technology Faculty of Mechatronics Institute of Metrology and Biomedical Engineering Warsaw Poland

**Keywords:** bone SPECT/CT, image quality, optimization, radiation dose

## Abstract

**Introduction:**

The purpose of this study was to present the optimization process of CT parameters to reduce patient exposure during bone SPECT/CT without affecting the quality of SPECT images with attenuation correction (AC).

**Material and methods:**

A fillable phantom reflecting realistic bone scintigraphy conditions was developed and acquired on an AnyScan SC. SPECT/CT scans were carried out with different x‐ray tube current values (10, 20, 30, 40, 50, 60, 70, 90, 110, 130, 150, and 200 mA) at three different high‐voltage values (80, 100, and 120 kV). The contrast (C) and coefficients of variation (CV) in the SPECT images as well as the signal‐to‐noise ratio (SNR) and noise (SD_CT_) in the CT images with CTDI_vol_ were measured. An optimal acquisition protocol that obtained SPECT/CT images with no artifacts on both CT and SPECT images, acceptable C, SNR, CV, and SD_CT_ values, and the largest reduction in patient exposure compared to the reference acquisition procedure was sought.

**Results:**

The optimal set of parameters for bone SPECT/CT was determined based on a phantom study. It has been implemented in clinical practice. Two groups of patients were examined according to the baseline and optimized protocols, respectively. The new SPECT/CT protocol substantially reduced patients’ radiation exposure compared to the old protocol while maintaining the required diagnostic quality of SPECT and CT images.

**Conclusions:**

In the study, we present a methodology that finds a compromise between diagnostic information and patient exposure during bone SPECT/CT procedures.

## 1 INTRODUCTION

Bone scintigraphy (whole body plus SPECT/CT) is one of the most common procedures in nuclear medicine practice. It visualizes the entire skeleton in a rapid and relatively low‐cost way to provide important diagnostic information, particularly in oncology.^1^, ^2^ Widespread use of SPECT/CT hybrid devices has undoubtedly improved the quality of patient care. However, an increasing number of diagnostic examinations performed with their help results in a significant increase in total effective dose to patients.^3^ While an effective SPECT dose mainly depends on the radiopharmaceutical activity injected into the patient and is usually an average level of 3–4 mSv, an effective CT dose can vary substantially depending on the device used, exposure parameters, and diagnostic center practice.^4^ For higher dose diagnostic quality CT studies, an effective dose may range between 4 and 14 mSv.^4^, ^5^, ^6^, ^7^ In CT scans performed to localize pathological tracer uptake found in SPECT and attenuation correction (AC) (low‐dose CT), effective doses in the range of 0.6 mSv–4 mSv were reported.^8^, ^9^ It is estimated that for ^99m^Tc‐MDP bone scintigraphy with SPECT/CT examination, even reduced exposure parameters may increase the total effective dose in the range of approximately 60–85% compared to SPECT without CT.^10^, ^11^ That is why many nuclear medicine specialists are interested in reducing patient exposure to ionizing radiation during SPECT/CT, especially emphasizing CT.^3^, ^12^, ^13^, ^14^, ^15^, ^16^ However, patient exposure during CT is directly related to CT image quality, which cannot lose its diagnostic value due to different exposure parameters. The selection of exposure parameters, mainly x‐ray tube current and high voltage, has a major impact on both aspects of CT examination. Excessively high values of these parameters do not necessarily lead to additional diagnostic information, but result in greater exposure of patients and staff. Lower values in turn minimize exposure, but can also lead to lower diagnostic CT image value. Two publications presenting studies on the optimization of the patient dose as well as the CT image quality as a part of bone SPECT/CT examination can be found in the literature.^17^, ^18^ In both articles, the authors compared only two sets of CT exposure parameters. In addition, both papers did not justify on what basis the CT parameter sets were selected for comparison.

The relationship between CT image quality and SPECT image quality through the AC procedure is also an important issue. AC of SPECT data using CT data must overcome fundamental difficulties: the polyenergetic characteristics of the continuous x‐ray energy spectrum in computed tomography and differences in the radiation energy used to obtain SPECT and CT images. It is therefore necessary to convert x‐ray linear attenuation coefficient µ (associated with Hounsfield units [HU]) to the µ coefficient of gamma radiation (140 keV for ^99m^Tc). AC methods have their limitations, especially in dense materials.^19^, ^20^, ^21^ Bone tissue is a mineralized structure with two tissue types: cortical bone and cancellous bone. Cortical bone has a HU value of approximately 1,700–2,000, whereas cancellous bone has a HU value of approximately 150–300.^22^ In addition, beam hardening from a dense CT target may affect the determination of the μ coefficient for an object’s given element depending on its location in that object. Most CT scanners have implemented procedures to correct beam hardening, working effectively for tissues similar in density to water. However, algorithms may behave less accurately for high‐density structures (that is, bones or implants).^23^ It was also shown that the noise level in SPECT images reconstructed using the MLEM iterative method is proportional to the noise level in corresponding μ maps.^24^ The noise level in CT images can also affect SPECT image quality by lowering the local µ coefficient values.^25^, ^26^ Noise and artifacts in CT images can potentially decrease SPECT image quality during the AC procedure and may also have a negative impact on its anatomical assessment.^26^ All of these issues may have an impact on SPECT/CT image quality and inaccurate determination of µ maps used for AC. They can lead to artifacts or other errors in SPECT images, especially in bone imaging using ^99m^Tc‐MDP, which binds to high‐density tissue. It can be postulated for bone SPECT/CT that the lowest effective CT dose (and the corresponding lowest values of exposure parameters) will be achieved when the impact of low‐dose CT on reconstructed SPECT becomes practically unacceptable.^26^

The purpose of this study was to optimize CT parameters to reduce patient exposure during bone SPECT/CT without affecting the quality of SPECT images with AC.

## 2 MATERIALS AND METHODS

### 2.A Stage I: Phantom study

#### 2.A.1 Phantom

**Fig. 1:**
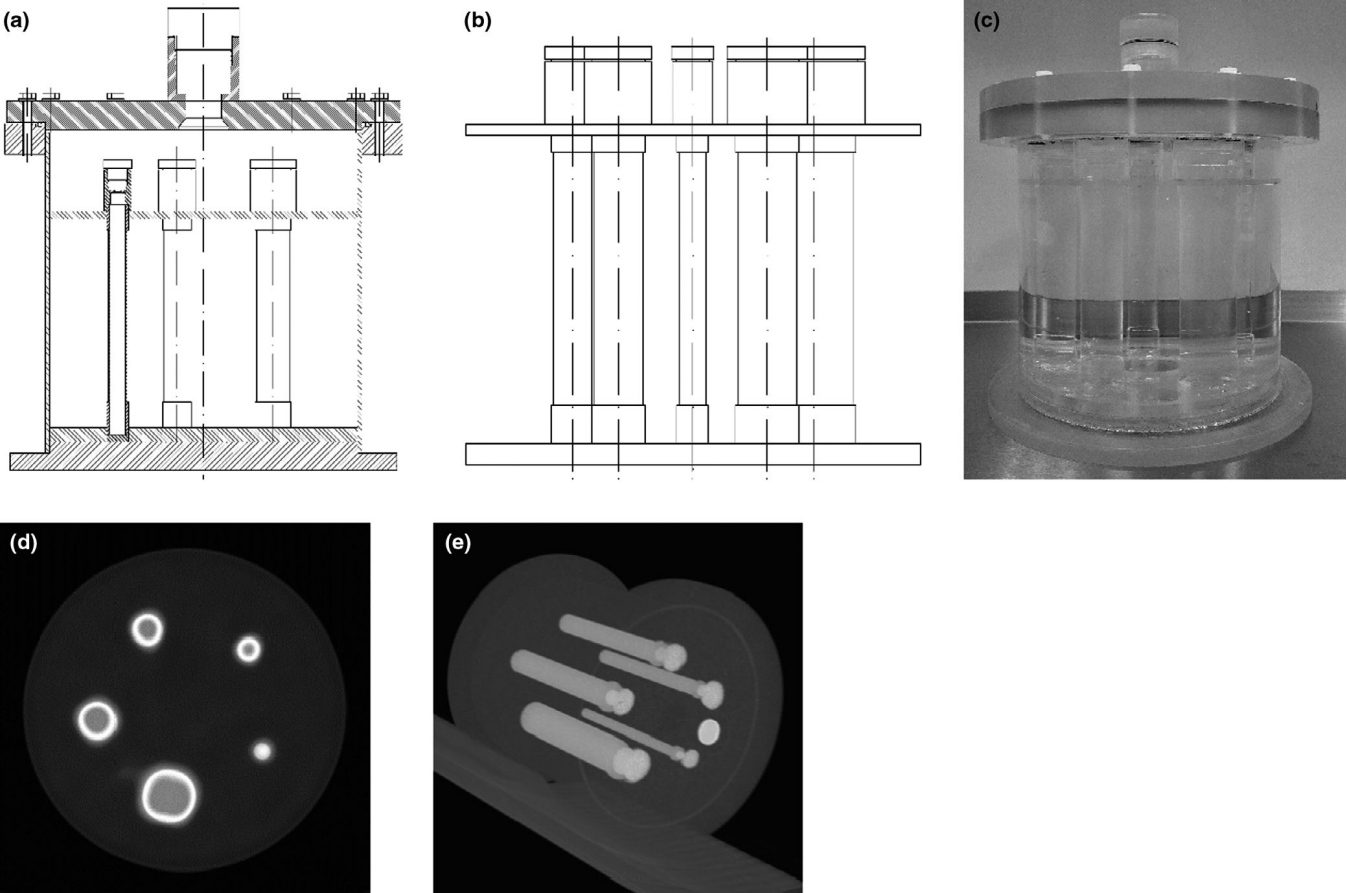
The phantom. (a) Schematic drawing of the phantom body with insert. (b) Schematic drawing of the insert itself. (c) Photo of the phantom. (d) SPEC/CT image. (e) MIP CT image.

A fillable phantom reflecting realistic bone scintigraphy conditions were developed and constructed (Fig. 1). The phantom body was a source tank with a protruding flanged polymethyl methacrylate (PMMA) top with a 3‐mm wall thickness, 21.5 cm interior diameter, and 22.5 cm interior height. An insert consisting of five PMMA cylinders in a pie arrangement was placed in the source tank. Cylinders simulating long bones with various sizes had interior diameters of 11 mm, 16 mm, 21 mm, 26 mm, and 36 mm, respectively.

The phantom body was filled with water mixed with sodium pertechnetate solution Na^99m^TcO_4_ (a warm background simulating residual activity in a patient's body outside the skeleton). Cylinders (hot sources simulating accumulation of the main pool of activity in the patient's skeleton) were filled with a solution of di‐potassium hydrogen phosphate (K_2_HPO_4_) mixed with water and Na^99m^TcO_4_ (100 g of salt was dissolved in 67 g of water).^27^ It was shown in Ref. 27 that the high solubility of K_2_HPO_4_ salt and its elemental weight composition allows the preparation of mixtures simulating bone attenuation. The ratio of the activity concentration in hot sources to the background was 10:1 (hot sources 50 kBq/ml and background 5 kBq/ml). The insert with the smallest diameter had a HU value of 1100 ± 250. The insert with the largest diameter had a HU value of 1250 ± 170.

#### 2.A.2 Image acquisition and reconstruction

All of the measurements were conducted using an AnyScan SC (Mediso Medical Imaging Systems, Budapest, Hungary) equipped with dual‐head SPECT gamma camera (3/8” NaI(Tl) crystal, 60 photomultiplier tubes, and 54.5 × 40.5 cm field of view) and multidetector CT (16 rows). The SPECT/CT scanner was installed at the Nuclear Medicine Department of Maria Sklodowska‐Curie National Research Institute of Oncology, Krakow Branch, in 2009.

First, a phantom image was acquired with the standard SPECT/CT protocol used at the Nuclear Medicine Department. SPECT acquisition was performed at the following settings: low‐energy high‐resolution (LEHR) parallel collimators, 128 × 128 matrix, 4.14 mm pixel size, non‐circular orbit, step‐and‐shoot mode with 32 projection angles acquired over 360° and 20 s per projection, two energy windows (140 keV ± 7.5% for ^99m^Tc photopeak, 120 keV ± 7.5% for Compton down‐scatter). The SPECT images were reconstructed using an ordered‐subset expectation maximization iterative reconstruction algorithm (OSEM) with four subsets, 10 iterations with attenuation correction (CT data were used to create attenuation‐correction maps), scatter correction (dual‐energy window method), and resolution recovery correction. Unenhanced CT scans were obtained using helical rotation (x‐ray tube high voltage 120 kV, tube current 50 mA, primary beam collimation 20 mm, rotation 1 s, pitch 1.0, and axial field‐of‐view 50 cm) and reconstructed using a 512 x 512 matrix, slice thickness of 2.5 mm, filtered back projection method (FBP), convolution kernel recommended by the manufacturer, high resolution, and beam‐hardening corrections. The angular variation of the x‐ray tube current was not available. The final SPECT/CT image was considered as a baseline in further analysis (the reference image). Second, a series of CT scans was carried out with different x‐ray tube current values (10, 20, 30, 40, 50, 60, 70, 90, 110, 130, 150, and 200 mA) at three different high voltage values (80, 100, and 120 kV). Each CT was utilized for the AC of the SPECT data acquired with baseline parameters. A total of 36 SPECT/CT images were obtained in a single measurement series for further analysis. This experiment was repeated three times (3 measurement series). The phantom was refilled between measurement series to maintain a comparable activity concentration.

#### 2.A.3 Image evaluation

All of the evaluations were conducted on a workstation with an InterView Fusion application (Mediso Medical Imaging Systems, Budapest, Hungary) allowing fusion of the images and their volumetric analysis.

For the qualitative analysis, the SPECT/CT images were visually assessed by two observers in a masked manner. The SPECT images were analyzed using the spectrum color scale. The CT images were analyzed in grayscale (2000/400 HU window cylinders, 350/40 HU window background). The image quality was scored on a 3‐point scale: 2 = no visible artifacts in CT image, no noticeable deterioration of background uniformity in SPECT image, and all five hot sources visible; 1 = streak artifacts slightly visible in CT image and noticeable deterioration of background uniformity or at least four hot sources visible in SPECT image, 0 = streak artifacts clearly visible in CT image and a significant deterioration of background uniformity and hot sources visibility in SPECT image (at least three hot sources still visible).

For the quantitative analysis, the performance parameters were determined using the cylindrical volumes of interest (VOIs). VOIs were initially created on the CT images and then copied onto the corresponding SPECT images. For each hot source, three VOIs were delineated: in the center (1/2), in 1/4, and in 3/4 of the cylinder height. The VOI diameter corresponded to each cylinder’s diameter. The background region was defined by five VOIs (approximately 5 cm^3^ each) in the middle of the phantom between hot sources. For each reconstructed SPECT image, the contrast in the i‐th hot source (C_i_) was calculated using equation 1.

(1) $$C_{i}=\left({\text{Ns}}_{i}‐\text{Nb}\right)/\text{Nb}$$

where Ns_i_ is the total number of counts per mL in the i‐th hot source VOI and Nb is the mean total number of counts per mL in five background VOIs. To measure the SPECT image noise, the coefficient of variation (CV) was calculated using equation 2.

(2) $$\text{CV}={\text{SDb}}_{\text{SPECT}}/\text{Nb}$$

where SDb_SPECT_ is the mean standard deviation of counts per mL in five background VOIs.

For each CT image, the signal‐to‐noise ratio in the i‐th hot source (SNR_i_) was calculated in relation to the background according to equation (3).

(3) $${\text{SNR}}_{i}={\text{HU}}_{i}/{\text{SD}}_{\text{CT}}$$

where HUi corresponds to the mean reconstructed HU value in the i‐th hot‐source and SD_CT_ is the mean standard deviation of the pixel value in five background VOIs. The CT image noise was defined by SD_CT_.

##### 2.A.4 CT dose assessment

The volumetric CT dose index (CTDI_vol_) was determined to estimate the CT radiation exposure. Although the CTDI_vol_ values were automatically documented in a dose report, it was measured for each combination of CT parameters using a standardized CTDI_vol_ body phantom (32 cm) and a calibrated dose meter (X2 base unit with an X2 CT sensor, which was a pencil chamber including an electrometer, RaySafe X2, Unfors RaySafe AB, Billdal, Sweden).

#### 2.A.5 Optimization methodology

The optimal set of parameters for bone SPECT/CT was selected in the following way:

1. Visual assessment of the SPECT/CT images: exclusion of CT parameter sets for which the reconstructed SPECT/CT images were visually scored lower than the reference (defined as a phantom image acquired with the standard SPECT/CT protocol used at the Nuclear Medicine Department described in detail in Section 2.A.2).
2. Quantitative assessment of the SPECT/CT images: exclusion of CT parameter sets for which the mean C and SNR values in at least two hot sources were significantly lower or the mean CV and SD_CT_ values were higher than the reference.
3. Selection of the optimal set of exposure parameters corresponding to the lowest CTDI_vol_ value.

### 2.B Stage II: Clinical study

#### 2.B.1 Patients

Patients’ images included in the study were obtained between December 2017 and June 2018 at the Nuclear Medicine Department of Maria Sklodowska‐Curie National Research Institute of Oncology, Krakow Branch. All of the patients provided written informed consent for ^99m^Tc‐MDP bone examination. SPECT/CT of the abdominal part of the skeleton was performed on all of the subjects. Patients were examined according to the baseline SPECT/CT protocol from December 1, 2017, to April 6, 2018. Patients were examined according to the new optimal SPECT/CT protocol from April 7, 2018, to June 15, 2018. The local research ethics committee waived the need for formal approval because both SPECT/CT protocols were not used in the same patients. Additionally, according to national law,^28^ the physician (the nuclear medicine specialist) responsible for the examination had clinical liability in particular for justification of medical exposure and optimization of radiological protection. All of the examinations were conducted as part of routine diagnostics after consulting a nuclear medicine specialist.

#### 2.B.2 Image processing and evaluation

The SPECT/CT examination was conducted immediately after whole‐body examination approximately 2.5 hours after intravenous administration of ^99m^Tc‐MDP with a maximum activity of 740 MBq. The noncontrast CT was performed for AC and anatomical localization purposes only.

Images obtained using the two SPECT/CT protocols were compared: the baseline (old) and the optimized protocol (new). They differed only by x‐ray tube current (old 50 mA vs new 40 mA). Other acquisition, exposure, and reconstruction parameters were set as described in a phantom study. The same SPECT/CT scanner and processing station were used for the quantitative and qualitative image analyses as in I stage of the study. The SPECT/CT image assessment was based on methodology described in Ref. 17. For the qualitative analysis, the SPECT/CT images were visually assessed by two observers in a masked manner. The SPECT and CT images were analyzed as previously described in spectrum color scale and grayscale, respectively. The image quality was scored on a 5‐point Likert scale (5 = excellent to 1 = unacceptable).^29^ Quantitative SPECT and CT assessment was carried out via the volumetric analysis of two cylindrical VOIs. VOIs (both approximately 1 cm^3^) were placed in the area of the third lumbar vertebra (L) and in the corresponding region in the abdominal aorta (A). The total numbers of N and SD_SPECT_ counts within the VOIs in the SPECT images and the average density HU and SD_CT_ in the CT images were determined. The contrast (C) in the SPECT images was calculated as follows:

(4) $$C=\left(N_{L}‐N_{A}\right)/N_{A}$$

For the aorta VOI, the coefficient of variation (CV_A_) in the SPECT images was determined according to equation:

(5) $${\text{CV}}_{A}={\text{SD}}_{\text{SPECTA}}/N_{A}$$

The SNR in the CT images was calculated analogous to equation (3) as follows:

(6) $$\text{SNR}={\text{HU}}_{A}/{\text{SD}}_{\text{CTA}}$$

The CT image noise was defined by SD_CTA_.

#### 2.B.3 CT dose assessment

Patient exposure was estimated in both groups according to a report by the American Association of Physicists in Medicine Task Group (AAPM TG) 204.^30^ A size‐specific dose estimate (SSDE) was calculated for each patient. The sum of the anterior–posterior and lateral dimensions on abdominal CT (a surrogate for patient size) was used to scale CTDI_vol_ automatically reported by the scanner. The method based only on patient geometry and did not consider the different attenuation of various tissue types as reported in AAPM Report 220.^31^ The new report recommended the use of the water‐equivalent diameter (D_w_), which considers tissue attenuation in addition to patient geometric size. However, the errors resulting from using a patient size‐corrected dose estimate only were in the range of a few percent in the abdominal region.^31^ Thus, we used the SSDE based on any of the geometric data in accordance with AAPM Report 204.

### 2.C Statistics

In study stage I, Welch’s t‐test was used to compare the mean C, CV, SNR, and SD_CT_ values determined for the reference image and images obtained using the exposition parameter sets. In study stage II, the paired Wilcoxon/Mann–Whitney test or Student's t‐test was used to compare two independent groups depending on the data distribution. The Shapiro–Wilk method was used to test the data set distribution. A p value of less than 0.05 was considered statistically significant.

## 3 RESULTS

### 3.A Stage I: Phantom study

#### 3.A.1 Qualitative analysis

The results of the visual assessment of the phantom SPECT/CT images obtained for various combinations of CT exposure parameters are presented in Table 1.

**Table 1:** Visual assessment of the phantom SPECT/CT images.

High voltage [kV]	X‐ray tube current [mA]																							
	10		20		30		40		50		60		70		90		110		130		150		200	
	C	S	C	S	C	S	C	S	C	S	C	S	C	S	C	S	C	S	C	S	C	S	C	S
80	0	1	0	2	0	2	0	2	1	2	1	2	1	2	2	2	2	2	2	2	2	2	2	2
100	0	1	0	2	1	2	1	2	2	2	2	2	2	2	2	2	2	2	2	2	2	2	2	2
120	1	2	1	1	2	2	2	2	RI		2	2	2	2	2	2	2	2	2	2	2	2	2	2

C, CT part of the SPECT/CT image; S, SPECT part of the SPECT/CT image; RI, the reference image.

#### 3.A.2 Quantitative analysis

Detailed results (mean values with standard deviation of C and SNR for hot sources and CV and SD_CT_ in the reconstructed images for each analyzed parameter sets) are presented in Supplementary A (Table A1 and Table A2).

**Fig. 2:**
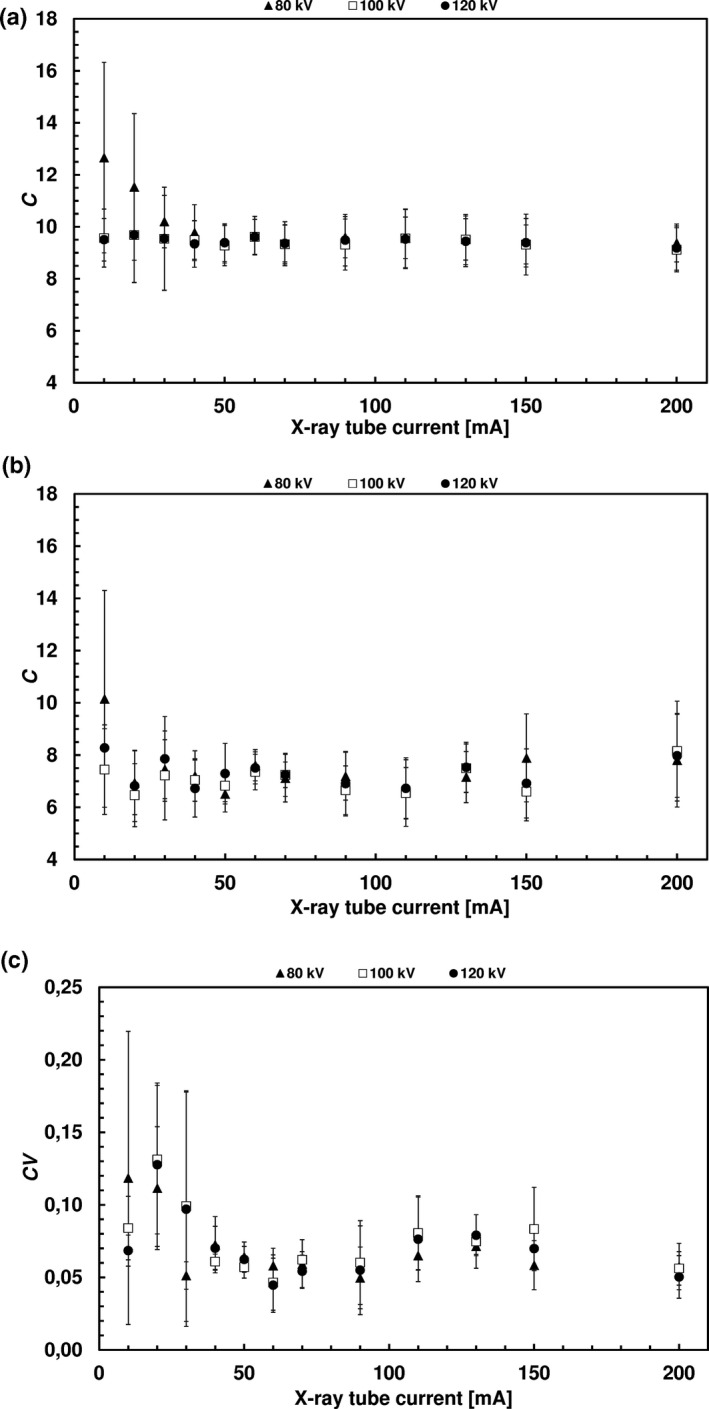
SPECT images. (a) Mean C value of the hot source with the largest diameter. (b) Mean C value of the hot source with the smallest diameter. (c) Mean CV.

**Fig. 3:**
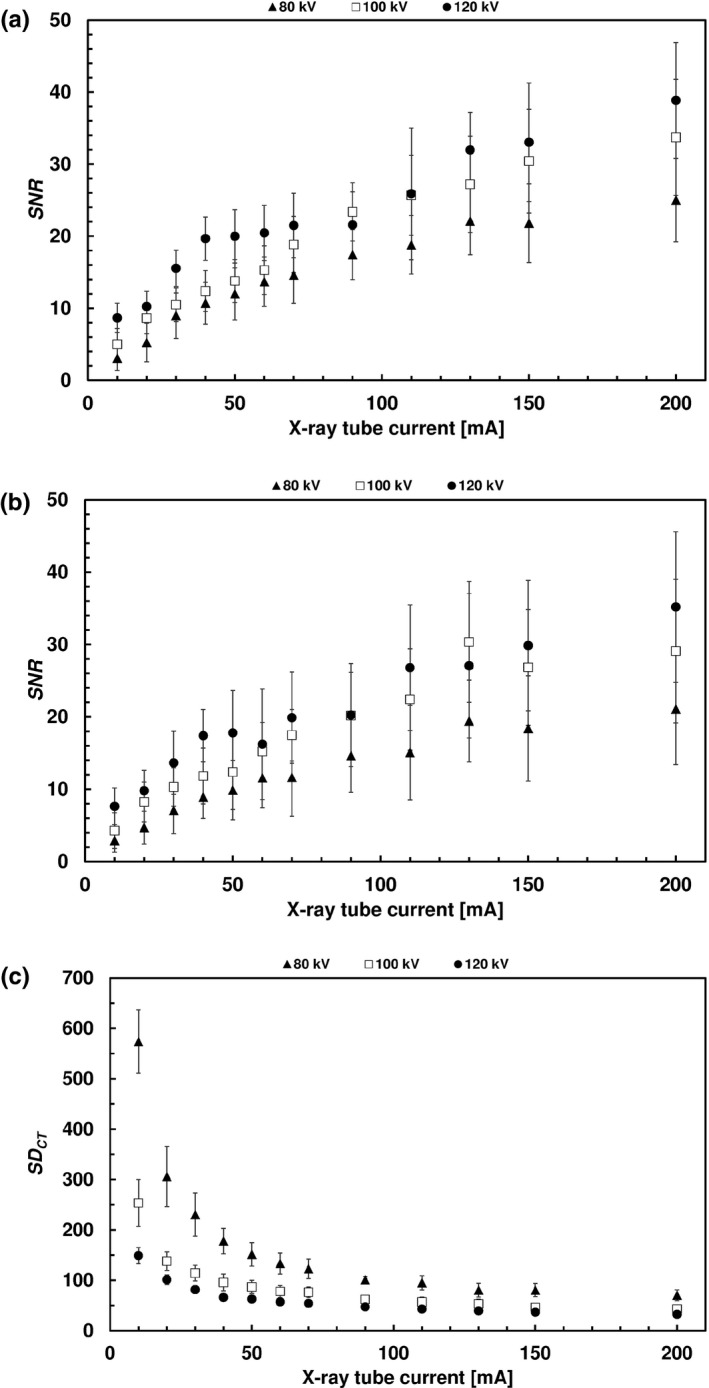
CT images. (a) Mean SNR value of the hot source with the largest diameter. (b) Mean SNR value of the hot source with the smallest diameter. (c) Mean SDCT.

Fig. 2 and 3 present the C and SNR values obtained for two hot sources (with the largest and smallest diameters). The CV and SD_CT_ values are also shown. The measuring points correspond to the mean group values and the bars correspond to the SD.

Table 2 presents the volumetric CT dose index CTDI_vol_ values that estimated ionizing radiation exposure during CT scanning.

**Table 2:** CT dose index (CTDI_vol_ [mGy], (%)). The CTDI_vol_ value of the reference image was set to 100%.

X‐ray tube current [mA]	High voltage [kV]		
	80	100	120
10	0.6 (7%)	1.2 (13%)	1.9 (21%)
20		2.3 (25%)	3.6 (41%)
30	1.7 (19%)	3.1 (35%)	5.4 (60%)
40			7.1 (80%)
50	2.8 (32%)	5.5 (62%)	8.9 (100%) RI
60			10.6 (119%)
70	3.8 (43%)	7.4 (84%)	11.9 (134%)
90	4.9 (56%)	9.6 (108%)	15.3 (173%)
110	6.1 (68%)	11.8 (133%)	19.0 (214%)
130	7.2 (81%)	14.0 (157%)	22.4 (252%)
150	8.3 (93%)	16.2 (182%)	25.9 (292%)
200	10.9 (123%)	21.3 (240%)	34.1 (385%)

RI, reference image.

#### 3.A.3 Optimization of bone SPECT/CT protocol

The decision strategy presenting the next optimization steps according to the adopted criteria are presented in Table 3. The elimination criteria were as follows:

**Table 3:** The decision strategy for finding an optimal image corresponding to the optimal CT parameters.

X‐ray tube current [mA]	High voltage [kV]		
	80	100	120
10	V, S, *σ*	V, S, *σ*	V, S, *σ*
20	V, S, *σ*	V, S, *σ*	V, S, *σ*
30	V, S, *σ*	V, S, *σ*	S, *σ*
40	V, S, *σ*	V, S	OI
50	V, S, *σ*	S, *σ*	RI
60	V, C, S, *σ*	*C, σ*	D
70	V, S, *σ*	*σ*	D
90	S, *σ*	D	D
110	*σ*	D	D
130	*σ*	D	D
150		D	D
200	*D*	D	D

RI, reference image; OI, optimal image.

V: eliminated based on the visual assessment (a score of 0 or 1 for at least one of the SPECT or CT images).

C: eliminated based on the quantitative assessment of contrast C (a significantly lower mean C value for at least two hot sources compared to the reference image).

CV: eliminated based on the quantitative assessment of the coefficient of variation CV (a significantly higher mean CV value than the reference image).

S: eliminated based on the quantitative assessment of the signal‐to‐noise ratio (SNR) (a significantly lower mean SNR value for at least two hot sources than the reference image).

*σ*: eliminated based on the quantitative assessment of the noise SD_CT_ (a significantly higher mean SD_CT_ value than the reference image).

D: eliminated based on the quantitative assessment of the radiation exposure (a higher CTDI_vol_ than the reference image).

### 3.B Stage II: Clinical study

#### 3.B.1 Patients

**Fig. 4:**
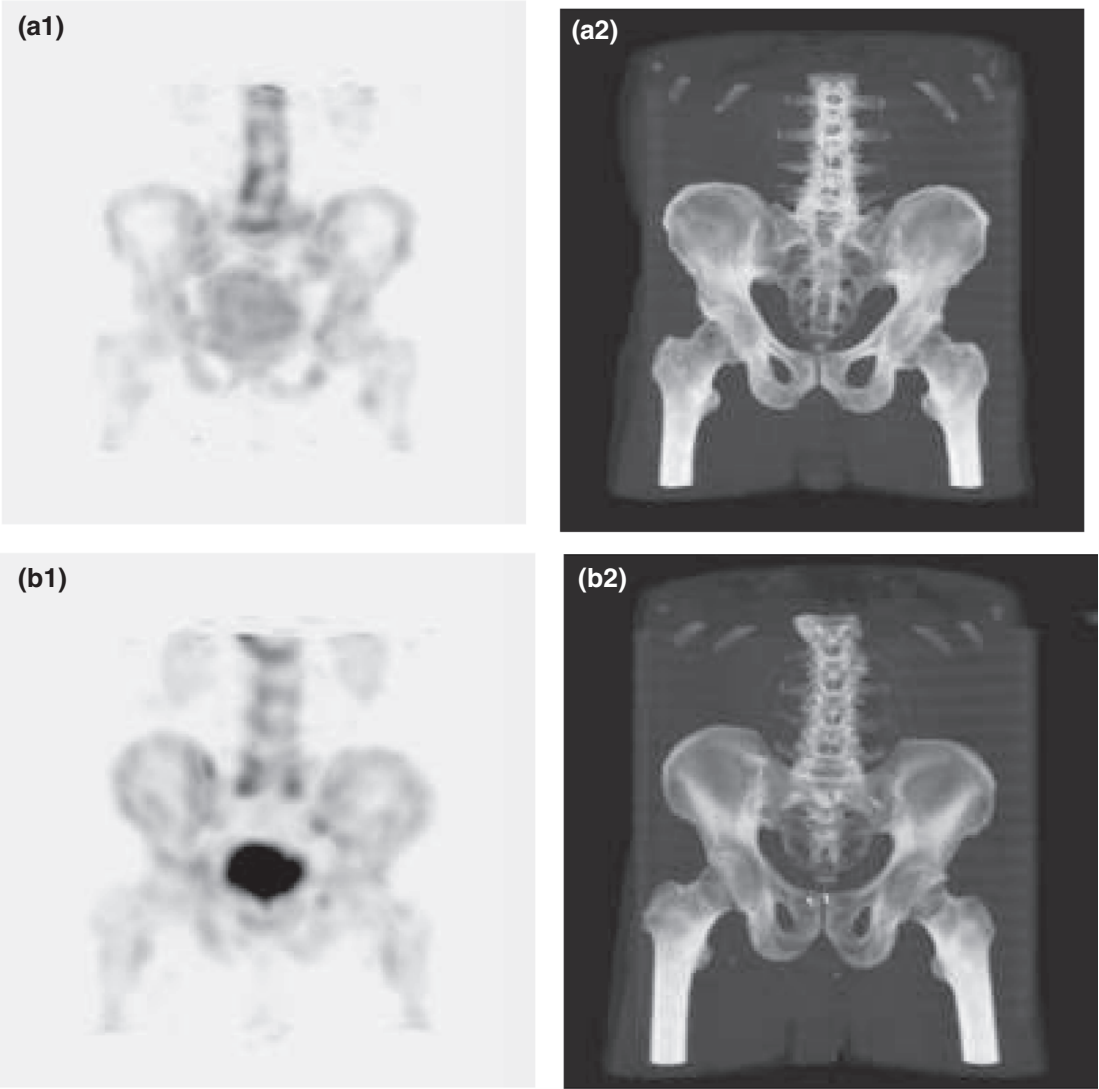
Patient from group I: a1. SPECT, a2. CT. Patient from group II: b1. SPECT, b2. CT.

In 68 patients, bone SPECT/CT was prospectively performed for staging and follow‐up. All of the patients (except one) were referred for bone scintigraphy because of cancer. The primary diagnosis was: breast cancer (n = 44 [65%]), prostate cancer (n = 8 [13%]), gynecological cancer (n = 4 [7%]), unknown primary carcinoma (n = 3 [5%]), sarcoma (n = 2 [3%]), kidney cancer (n = 2 [3%]), lung cancer (n = 1 [1%]), bladder cancer (n = 1 [1%]), melanoma (n = 1 [1%]), and lymphoma (n = 1 [1%]). One patient had a nononcological disease (coxarthrosis). The study population was divided into two groups. The first group (group I: 28 women and 7 men) was examined according to the baseline (old) SPECT/CT protocol. The second group (group II: 27 women and 6 men) was examined according to the optimized (new) SPECT/CT protocol. The two groups’ characteristics were comparable in terms of age, weight, height, and body mass index (BMI) (Table 4). Fig. 4 shows the SPECT and CT MIP images obtained using the old and optimized protocol from two representative patients.

**Table 4:** Age, weight, height, and BMI.

	Group	Min	Max	Median	*P*
Age [y]	I	40	85	70 mean 67 ± 11	0.078**
	II	33	86	63 mean 62 ± 13	
Weight [kg]	I	53	110	70	0.932*
	II	50	102	71	
Height [m]	I	1.5	1.8	1.6	0.430*
	II	1.5	1.8	1.6	
BMI [kg/m^2^]	I	21	40	26	0.638*
	II	19	36	26	

* Paired Wilcoxon/Mann–Whitney test.

** Student's *t*‐test.

#### 3.B.2 Qualitative analysis

The CT and SPECT image quality between groups I and II was not significantly different, with an equal median Likert score of 3 (min 2, max 4) in both techniques and groups. The *P* value was equal to 0.401 and 0.142 in the groups’ SPECT and CT images, respectively.

#### 3.B.3 Quantitative analysis

The quantitative SPECT evaluation showed no difference in contrast C between the groups, with a median C of 6.6 (min 2.1, max 18.6) for the old protocol and 7.1 (min 2.8, max 19.3) for the optimized protocol. However, a median CV_A_ of 0.3 (min 0.2, max 0.7) for group I was significantly lower than group II (median 0.4, min 0.2, max 0.7). The quantitative CT evaluation showed no difference in SNR and SD_CTA_ between the groups, with a median SNR of 2.1 (min 1.2, max 3.4) for the old protocol and 1.9 (min 1.2, max 6.5) for the optimized protocol and a median SD_CTA_ of 22.1 (min 13.4, max 41.5) for the old protocol and 22.4 (min 8.6, max 38.3) for the optimized protocol. The mean SSDE for group I (8 ± 1 mGy) was significantly higher than for group II (6 ± 1 mGy). The percentage difference between the analyzed mean values was 25% (Table 5).

**Table 5:** SPECT image contrast and noise, CT image SNR and noise, and CT radiation dose.

		Group	Min	Max	Median	*P*
SPECT	C	I	2.1	18.6	6.6	0.503*
		II	2.8	19.3	7.1	
	CV_A_	I	0.2	0.7	0.3	0.039*
		II	0.2	0.7	0.4	
CT	SD_CTA_	I	13.4	41.5	22.1	0.981*
		II	8.6	38.3	22.4	
	SNR	I	1.2	3.4	2.1	0.925*
		II	1.2	6.5	1.9	
SSDE [mGy]		I	5	10	7 mean 8 ± 1	<0.0001**
		II	5	8	6 mean 6 ± 1	

* Paired Wilcoxon/Mann–Whitney test.

** Student's *t*‐test.

## 4 DISCUSSION

Optimization of diagnostic procedures using ionizing radiation should be dictated by ALARA radiation protection principle (as low as reasonably achievable). In the context of this study, ALARA means the lowest exposure that leads to acceptable image quality for the vast majority of patients. The process of selecting optimal acquisition and exposure parameters (both in terms of image quality and patient exposure) should be carried out in a manner tailored to each clinical problem. This study proposes a two‐step optimization methodology for bone SPECT/CT (stage I: a phantom study and stage II: a clinical study).

At the first stage, the concept of a fillable phantom reflecting realistic bone scintigraphy conditions (accumulation of radiopharmaceuticals in dense structures) was developed. To the best of our knowledge, only two publications presenting comparable solutions can be found in the literature. The first paper concerned a three‐dimensional brain phantom with bone structures maintaining a realistic head contour.^32^ In the second paper, the authors described a fillable torso phantom containing a material with a density corresponding to bone tissue.^33^ Using a specially constructed phantom allowed the detailed assessment of the impact of the exposure parameters on SPECT/CT images of dense structures, both in terms of quality (visual inspection) and quantity (defined measures of image quality). The best set of exposure parameters in terms of image quality and exposure was determined on this basis. In the vast majority of cases, no statistically significant difference was found in the average contrast and coefficient of variance between the analyzed groups with respect to the reference image. However, attention should be paid to increasing values of C and COV uncertainties at the lowest currents (<30 mA). This suggests that it is necessary to remain careful in the eventual selection of the exposure parameters. The relationships between SNR and exposure parameters were as expected. The same could be said about CT noise vs exposure parameters.

A SPECT/CT image characterized by a lack of artifacts on both CT and SPECT images, acceptable C, SNR, CV, and SD_CT_ values, and the largest reduction in exposure (20%) compared to the reference image was reconstructed for 120 kV and 40 mA. After carefully analyzing the stage I results, the head of the Nuclear Medicine Department decided to change the CT parameters of the bone SPECT/CT protocol from 120 kV and 50 mA to 120 kV and 40 mA without changing the SPECT parameters.

In the study’s second stage, the selected set of parameters was implemented into clinical practice. It was experimentally confirmed that using the new optimal protocol significantly reduced patient exposure while maintaining diagnostic image quality. Of note, the SPECT image noise (median CV value) was significantly higher in the group of patients examined with the new protocol. But this result was not reflected in the qualitative analysis, so the higher nominal SPECT noise was not of any clinical consequence.

This study had limitations that merit mention.

First, only one type of SPECT/CT device was used in this study. Thus, the selected set of parameters was optimal for the device used to conduct the measurements. All of the examinations should be repeated on other SPECT/CT devices in the future, but we presume that the developed methodology can be implemented on any SPECT/CT scanner.

Another issue is the fact that the device used in this study was installed in 2009. Therefore, it is not the latest generation device equipped with currently available highly advanced image reconstruction algorithms. However, the proposed methodology achieved at least a 20% reduction in patients’ exposure during CT for the bone SPECT/CT examination. The question may be asked whether similar optimization methodology using advanced reconstruction algorithms may further reduce patient exposure while maintaining expected image quality. The behavior of CT iterative reconstruction algorithms in soft tissue has been thoroughly studied.^34^ Researchers have also focused on iterative CT reconstruction of bone tissue in which the signal‐to‐noise ratio is naturally high. The benefits of iterative reconstruction may not be very significant, but it can be expected that their use is an opportunity to further reduce patient exposure.^35^, ^36^, ^37^ The optimal bone SPECT image reconstruction has been also studied. Prior studies recommended using the OSEM iterative method and highlighted its superiority over the FBP technique.^38^, ^39^, ^40^ However, different OSEM reconstruction parameters were used in each study cited (number of subsets and iterations with or without additional filtration). The optimal reconstruction parameters should be selected for each SPECT procedure taking into account individual preferences of nuclear medicine specialists analyzing diagnostic images at a given nuclear medicine department. The methodology proposed in this paper can be also appropriate for similar types of research studies. However, the reconstruction parameters can be changed and the reconstruction procedure itself can be repeatedly carried out at any time after SPECT/CT data registration. The selected examination protocol (acquisition and exposure parameters) cannot be changed during data acquisition. Raw acquired data cannot be recollected without exposing patients to additional radiation doses. Hence, the proper selection of the acquisition and exposure parameters is important for the optimal performance of any diagnostic procedure using ionizing radiation.

## 5 CONCLUSIONS

In this study, we presented a methodology that finds a compromise between diagnostic information and patient exposure during bone SPECT/CT. The new SPECT/CT protocol established was implemented into clinical practice. It has substantially reduced patient exposure compared to the old protocol while maintaining the required diagnostic quality of SPECT and CT images. A prototype of a new phantom reflecting realistic clinical bone scintigraphy conditions (direct binding of marker to high‐density tissue) was developed and constructed.

## CONFLICT OF INTEREST

No conflict of interest.

## Supporting information


**Table A1**. Mean (±SD) C and CV values in SPECT images for each analysed parameter set. Bold mean values are statistically significantly different from the reference.
**Table A2**. Mean (±SD) SNR and SD_CT_ values in CT images for each analysed parameter set. Bold mean values are statistically significantly different from the reference.
