# Supplementary material for: On the optimization of bone SPECT/CT in terms of image quality and radiation dose
Source: J Appl Clin Med Phys. 2020 Oct 27;21(11):237–46. doi: 10.1002/acm2.13069 (PMC7700938; doi:10.1002/acm2.13069)
Supplement: Supplementary file 1 — Table A1. Mean (±SD) C and CV values in SPECT images for each analysed parameter set. Bold mean values are statistically significantly different from the reference. Table A2. Mean (±SD) SNR and SDCT values in CT images for each analysed parameter set. Bold mean values are statistically significantly different from the reference. [file ACM2-21-237-s001.docx]

**Supplement A**

Table A1. Mean (± SD) C and CV values in SPECT images for each analysed parameter set. Bold mean values are statistically significantly different from the reference.

|  | | C | | | | | CV |
| --- | --- | --- | --- | --- | --- | --- | --- |
|  |  | Cylinder diameter [mm] | | | | |  |
| High voltage [kV] | X-ray tube current [mA] | 40 | 30 | 25 | 20 | 15 |  |
| 80 | 10 | 12.7 ± 3.7 | 10.8 ± 2.7 | 11.5 ± 3.4 | 12.2 ± 4.2 | 10.2 ± 4.2 | 0.12 ± 0.10 |
|  | 20 | 11.5 ± 2.8 | 9.3 ± 1.7 | 9.6 ± 2.0 | 8.8 ± 1.8 | 6.9 ± 1.2 | 0.11 ± 0.04 |
|  | 30 | 10.2 ± 1.0 | 9.4 ± 0.7 | 8.8 ± 0.6 | 8.9 ± 1.1 | 7.5 ± 1.1 | 0.05 ± 0.01 |
|  | 40 | **9.8 ± 1.1** | 9.5 ± 1.0 | 9.0 ± 1.1 | 9.5 ± 1.5 | 7.2 ± 1.0 | 0.07 ± 0.02 |
|  | 50 | 9.4 ± 0.7 | 9.5 ± 0.8 | 8.9 ± 0.7 | 9.1 ± 1.4 | 6.5 ± 0.7 | 0.06 ± 0.01 |
|  | 60 | **9.7 ± 0.7** | **9.7 ± 0.7** | 9.4 ± 0.8 | 8.9 ± 0.9 | 7.6 ± 0.6 | 0.06 ± 0.02 |
|  | 70 | 9.3 ± 0.8 | 9.3 ± 0.9 | 9.2 ± 0.9 | 9.1 ± 1.5 | 7.1 ± 0.9 | 0.06 ± 0.03 |
|  | 90 | 9.6 ± 0.8 | 9.7 ± 1.3 | 9.3 ± 1.0 | 8.8 ± 0.8 | 7.2 ± 0.9 | 0.05 ± 0.04 |
|  | 110 | **9.6 ± 0.8** | 9.5 ± 0.8 | 9.1 ± 0.9 | 9.6 ± 1.0 | 6.5 ± 1.3 | 0.07 ± 0.02 |
|  | 130 | **9.5 ± 0.8** | 9.5 ± 0.8 | 9.2 ± 0.9 | 8.5 ± 0.8 | 7.2 ± 1.0 | 0.07 ± 0.01 |
|  | 150 | **9.3 ± 0.8** | 9.5 ± 0.8 | 9.3 ± 0.9 | 9.0 ± 1.1 | 7.9 ± 1.7 | 0.06 ± 0.02 |
|  | 200 | 9.4 ± 0.7 | **9.7 ± 0.8** | 9.4 ± 0.9 | 9.3 ± 1.2 | 7.8 ± 1.8 | 0.06 ± 0.02 |
| 100 | 10 | **9.6 ± 1.1** | 9.5 ± 1.4 | 8.8 ± 1.0 | 9.4 ± 1.6 | 7.4 ± 1.7 | 0.08 ± 0.03 |
|  | 20 | 9.7 ± 1.8 | 9.6 ± 1.8 | 9.5 ± 2.1 | 9.1 ± 1.7 | 6.5 ± 1.2 | 0.13 ± 0.05 |
|  | 30 | **9.5 ± 2.0** | 9.3 ± 2.0 | 9.3 ± 2.2 | 9.2 ± 2.0 | 7.2 ± 1.7 | 0.10 ± 0.08 |
|  | 40 | 9.5 ± 0.7 | 9.5 ± 0.7 | 8.6 ± 0.6 | 9.3 ± 1.0 | 7.0 ± 0.8 | 0.06 ± 0.01 |
|  | 50 | 9.3 ± 0.8 | 9.3 ± 0.8 | 9.0 ± 1.0 | 9.5 ± 1.0 | 6.8 ± 0.6 | 0.06 ± 0.01 |
|  | 60 | **9.6 ± 0.7** | **9.2 ± 0.7** | 8.7 ± 0.9 | 9.3 ± 0.9 | 7.4 ± 0.7 | 0.05 ± 0.02 |
|  | 70 | 9.3 ± 0.8 | 9.4 ± 0.8 | 9.0 ± 0.7 | 8.8 ± 1.0 | 7.2 ± 0.7 | 0.06 ± 0.01 |
|  | 90 | 9.3 ± 1.0 | **10.2 ± 1.1** | 9.3 ± 1.0 | 8.9 ± 1.3 | 6.7 ± 0.9 | 0.06 ± 0.03 |
|  | 110 | **9.6 ± 1.1** | 9.3 ± 1.1 | 8.9 ± 1.2 | 8.7 ± 1.6 | 6.5 ± 1.0 | 0.08 ± 0.03 |
|  | 130 | **9.5 ± 1.0** | 9.4 ± 1.0 | 9.0 ± 1.0 | 9.0 ± 1.0 | 7.5 ± 0.9 | 0.07 ± 0.02 |
|  | 150 | 9.3 ± 1.2 | 9.4 ± 1.2 | 9.1 ± 1.2 | 9.2 ± 1.2 | 6.6 ± 1.1 | 0.08 ± 0.03 |
|  | 200 | 9.1 ± 0.8 | 9.3 ± 0.9 | 9.1 ± 1.0 | 9.0 ± 1.2 | 8.2 ± 1.9 | 0.06 ± 0.01 |
| 120 | 10 | **9.5 ± 0.8** | 9.6 ± 1.1 | 8.8 ± 0.8 | 8.9 ± 1.1 | 8.3 ± 0.7 | 0.07 ± 0.01 |
|  | 20 | 9.7 ± 1.8 | 9.4 ± 1.9 | 9.6 ± 2.0 | 8.6 ± 1.9 | 6.8 ± 1.4 | 0.13 ± 0.06 |
|  | 30 | **9.5 ± 2.0** | 9.4 ± 2.0 | 9.2 ± 2.3 | 9.0 ± 2.1 | 7.9 ± 1.6 | 0.10 ± 0.08 |
|  | 40 | 9.3 ± 0.9 | 9.4 ± 0.9 | 8.6 ± 0.8 | 8.8 ± 1.3 | 6.7 ± 1.1 | 0.07 ± 0.02 |
| Reference | 50 | 9.4 ± 0.7 | 9.5 ± 0.8 | 9.1 ± 0.9 | 8.9 ± 1.3 | 7.3 ± 1.2 | 0.06 ± 0.01 |
|  | 60 | **9.6 ± 0.7** | 9.5 ± 0.7 | 8.8 ± 0.9 | 8.7 ± 0.8 | 7.5 ± 0.6 | 0.04 ± 0.02 |
|  | 70 | 9.4 ± 0.7 | **9.2 ± 0.7** | 9.0 ± 0.6 | 8.3 ± 0.8 | 7.2 ± 0.5 | 0.05 ± 0.03 |
|  | 90 | 9.5 ± 1.0 | 9.6 ± 1.4 | 8.9 ± 1.0 | 8.4 ± 1.0 | 6.9 ± 1.2 | 0.05 ± 0.03 |
|  | 110 | **9.5 ± 1.1** | 9.3 ± 1.1 | 8.8 ±1.1 | 9.0 ± 1.5 | 6.7 ± 1.2 | 0.08 ± 0.03 |
|  | 130 | 9.4 ± 1.0 | 9.6 ± 1.0 | 8.8 ± 0.9 | 8.7 ± 1.2 | 7.5 ± 1.0 | 0.08 ± 0.01 |
|  | 150 | 9.4 ± 0.9 | 9.2 ± 0.9 | 9.0 ± 0.9 | 9.1 ± 1.2 | 6.9 ± 1.3 | 0.07 ± 0.01 |
|  | 200 | 9.2 ± 0.8 | 9.3 ± 0.9 | 9.0 ± 1.1 | 8.9 ± 1.2 | 8.0 ± 1.6 | 0.05 ± 0.01 |

Table A2. Mean (± SD) SNR and SD_CT_ values in CT images for each analysed parameter set. Bold mean values are statistically significantly different from the reference.

|  | | SNR | | | | | SD_CT_ |
| --- | --- | --- | --- | --- | --- | --- | --- |
|  |  | Cylinder diameter [mm] | | | | |  |
| High voltage [kV] | X-ray tube current [mA] | 40 | 30 | 25 | 20 | 15 |  |
| 80 | 10 | **3.0 ± 1.7** | **3.0 ± 1.6** | **3.0 ± 1.4** | **2.8 ± 1.6** | **2.9 ± 1.6** | **574 ± 63** |
|  | 20 | **5.2 ± 2.7** | **5.0 ± 2.3** | **5.1 ± 2.0** | **4.9 ± 2.2** | **4.7 ± 2.3** | **306 ± 60** |
|  | 30 | **9.0 ± 3.2** | **8.3 ± 2.9** | **8.1 ± 2.9** | **7.8 ± 2.7** | **7.1 ± 3.2** | **230 ± 43** |
|  | 40 | **10.7 ± 2.9** | **9.8 ± 3.2** | **9.7 ± 3.1** | **9.5 ± 2.6** | **8.9 ± 2.9** | **178 ± 25** |
|  | 50 | **12.0 ± 3.6** | **11.6 ± 3.3** | **11.3 ± 3.5** | **10.9 ± 3.3** | **9.9 ± 4.1** | **151 ± 23** |
|  | 60 | **13.7 ± 3.4** | **13.1 ± 3.6** | **13.0 ± 3.2** | **12.3 ± 3.6** | **11.6 ± 4.1** | **133 ± 21** |
|  | 70 | 14.6 ± 3.9 | 14.2 ± 3.3 | **14.0 ± 4.0** | 13.7 ± 3.4 | **11.7 ± 5.4** | **123 ± 19** |
|  | 90 | **17.5 ± 3** | **17.1 ± 3.2** | **17.0 ± 2.5** | **15.4 ± 4.8** | **14.6 ± 5.1** | **101 ± 7** |
|  | 110 | 18.8 ± 4.1 | 18.4 ± 3.9 | 18.0 ± 3.9 | 17.3 ± 4.1 | 15.1 ± 6.5 | **95 ± 14** |
|  | 130 | 22.0 ± 4.7 | 21.2 ± 4.5 | 20.8 ± 5.9 | 19.5 ± 6.3 | 19.4 ± 5.7 | **80 ± 14** |
|  | 150 | 21.8 ± 5.5 | 21.7 ± 4.8 | 21.1 ± 5.0 | 19.6 ± 6.6 | 18.4 ± 7.3 | 81 ± 13 |
|  | 200 | 25.0 ± 5.8 | 24.7 ± 5.5 | 24.3 ± 5.1 | 23.0 ± 6.2 | 21.1 ± 7.7 | 70 ± 11 |
| 100 | 10 | **5.0 ± 2.2** | **4.9 ± 1.9** | **5.0 ± 1.8** | **4.6 ± 2.2** | **4.3 ± 2.5** | **253 + 46** |
|  | 20 | **8.6 ± 2.2** | **8.7 ± 2.0** | **8.6 ± 2.2** | **8.5 ± 2.4** | **8.2 ± 2.7** | **138 ± 18** |
|  | 30 | **10.5 ± 2.3** | **10.4 ± 2.6** | **10.5 ± 2.5** | **10.4 ± 2.8** | **10.3 ± 2.6** | **114 ± 16** |
|  | 40 | 12.4 ± 2.9 | **12.5 ± 2.7** | **12.5 ± 2.8** | **12.1 ± 3.6** | **11.8 ± 3.9** | 96 ± 17 |
|  | 50 | **13.8 ± 3.0** | **13.8 ± 3.0** | **13.8 ± 3.4** | 13.7 ± 3.6 | **12.4 ± 5.2** | **87 ± 13** |
|  | 60 | 15.3 ± 3.4 | 15.4 ± 3.3 | 15.3 ± 3.9 | 14.9 ± 4.5 | 15.2 ± 4.0 | **78 ± 12** |
|  | 70 | 18.8 ± 3.9 | 18.5 ± 3.7 | 18.0 ± 4.0 | 17.3 ± 4.3 | 17.5 ± 3.6 | **76 ± 10** |
|  | 90 | 23.4 ± 4.1 | 23.1 ± 3.6 | 22.0 ± 5.0 | 21.8 ± 4.0 | 20.2 ± 6.0 | 62 ± 8 |
|  | 110 | 25.7 ± 5.6 | 25.0 ± 5.8 | 24.9 ± 5.1 | 23.3 ± 6.4 | 22.4 ± 7.0 | 57 ± 10 |
|  | 130 | 27.2 ± 6.7 | 32.2 ± 7.8 | 32.9 ± 9.1 | 31.1 ± 9.7 | 30.3 ± 8.4 | 53 ± 10 |
|  | 150 | **30.4 ± 7.2** | **29.8 ± 7.2** | **29.4 ± 6.7** | **26.4 ± 9.7** | **26.8 ± 8.0** | 46 ± 7 |
|  | 200 | 33.7 ± 8.1 | 32.8 ± 8.4 | 32.7 ± 7.3 | 29.4 ± 11.0 | 29.1 ± 9.9 | **43 ± 8** |
| 120 | 10 | **8.7 ± 2.0** | **8.3 ± 2.0** | **8.0 ± 2.2** | **7.8 ± 1.9** | **7.6 ± 2.6** | **149 ± 16** |
|  | 20 | **10.2 ± 2.1** | **10.1 ± 2.6** | **10.3 ± 2.4** | **10.1 ± 2.6** | **9.8 ± 2.8** | **101 ± 10** |
|  | 30 | **15.5 ± 2.5** | **15.0 ± 3.0** | **14.7 ± 3.0** | **14.1 ± 3.0** | **13.6 ± 4.4** | **82 ± 8** |
|  | 40 | 19.7 ± 3.0 | 19.1 ± 3.4 | 18.7 ± 3.1 | 19.1 ± 4.0 | 17.4 ± 3.6 | 66 ± 5 |
| Reference | 50 | 20.0 ± 3.7 | 19.4 ± 4.1 | 19.3 ± 4.8 | 18.2 ± 4.4 | 17.8 ± 5.9 | 63 ± 7 |
|  | 60 | 20.4 ± 3.8 | 20.4 ± 4.1 | 20.5 ± 4.4 | 20.0 ± 5.5 | 16.2 ± 7.6 | **57 ± 6** |
|  | 70 | 21.5 ± 4.5 | 21.5 ± 4.8 | 21.5 ± 4.9 | 20.9 ± 6.1 | 19.9 ± 6.3 | **54 ± 6** |
|  | 90 | **21.6 ± 4.6** | **21.7 ± 4.8** | **21.6 ± 5.1** | **21.6 ± 5.1** | **20.2 ± 7.1** | **47 ± 5** |
|  | 110 | 25.9 ± 9.1 | **27.0 ± 7.8** | **27.8 ± 7.7** | **27.4 ± 6.1** | 26.8 ± 8.7 | **43 ± 6** |
|  | 130 | **32.0 ± 5.2** | **31.5 ± 6.6** | **30.2 ± 6.8** | **28.1 ± 8.6** | 27.1 ± 10.0 | **39 ± 5** |
|  | 150 | **33.0 ± 8.2** | **32.9 ± 7.8** | **33.0 ± 8.0** | **32.1 ± 9.5** | **29.8 ± 9.0** | **37 ± 5** |
|  | 200 | **38.8 ± 8.0** | **38.2 ± 8.0** | **38.1 ± 7.2** | **36.2 ± 10.3** | **35.2 ± 10.4** | **32 ± 5** |
